# Supplementary material for: VEGF and Other Gene Therapies Improve Flap Survival—A Systematic Review and Meta-Analysis of Preclinical Studies
Source: Int J Mol Sci. 2024 Feb 23;25(5):2622. doi: 10.3390/ijms25052622 (PMC10932385; doi:10.3390/ijms25052622)
Supplement: Supplementary file 1 [file ijms-25-02622-s001.zip › ijms-2847345-supplementary.pdf]

Table S1. PRISMA checklist.

Section and Topic	Item #	Checklist item	Location where item is reported
**TITLE**			
Title	1	Identify the report as a systematic review.	1
**ABSTRACT**			
Abstract	2	See the PRISMA 2020 for Abstracts checklist.	2
**INTRODUCTION**			
Rationale	3	Describe the rationale for the review in the context of existing knowledge.	3-4
Objectives	4	Provide an explicit statement of the objective(s) or question(s) the review addresses.	3-4
**METHODS**			
Eligibility criteria	5	Specify the inclusion and exclusion criteria for the review and how studies were grouped for the syntheses.	5-8
Information sources	6	Specify all databases, registers, websites, organisations, reference lists and other sources searched or consulted to identify studies. Specify the date when each source was last searched or consulted.	5
Search strategy	7	Present the full search strategies for all databases, registers and websites, including any filters and limits used.	5 (available from PROS-PERO)
Selection process	8	Specify the methods used to decide whether a study met the inclusion criteria of the review, including how many reviewers screened each record and each report retrieved, whether they worked independently, and if applicable, details of automation tools used in the process.	6
Data collection process	9	Specify the methods used to collect data from reports, including how many reviewers collected data from each report, whether they worked independently, any processes for obtaining or confirming data from study investigators, and if applicable, details of automation tools used in the process.	6-7
Data items	10a	List and define all outcomes for which data were sought. Specify whether all results that were compatible with each outcome domain in each study were sought (e.g. for all measures, time points, analyses), and if not, the methods used to decide which results to collect.	6-7
	10b	List and define all other variables for which data were sought (e.g. participant and intervention characteristics, funding sources). Describe any assumptions made about any missing or unclear information.	6-7
Study risk of bias assessment	11	Specify the methods used to assess risk of bias in the included studies, including details of the tool(s) used, how many reviewers assessed each study and whether they worked independently, and if applicable, details of automation tools used in the process.	8

Section and Topic	Item #	Checklist item	Location where item is reported
Effect measures	12	Specify for each outcome the effect measure(s) (e.g. risk ratio, mean difference) used in the synthesis or presentation of results.	7-8
Synthesis methods	13a	Describe the processes used to decide which studies were eligible for each synthesis (e.g. tabulating the study intervention characteristics and comparing against the planned groups for each synthesis (item #5)).	7
	13b	Describe any methods required to prepare the data for presentation or synthesis, such as handling of missing summary statistics, or data conversions.	6-7
	13c	Describe any methods used to tabulate or visually display results of individual studies and syntheses.	7-8
	13d	Describe any methods used to synthesize results and provide a rationale for the choice(s). If meta-analysis was performed, describe the model(s), method(s) to identify the presence and extent of statistical heterogeneity, and software package(s) used.	7-8
	13e	Describe any methods used to explore possible causes of heterogeneity among study results (e.g. subgroup analysis, meta-regression).	7-8
	13f	Describe any sensitivity analyses conducted to assess robustness of the synthesized results.	7-8
Reporting bias assessment	14	Describe any methods used to assess risk of bias due to missing results in a synthesis (arising from reporting biases).	8
Certainty assessment	15	Describe any methods used to assess certainty (or confidence) in the body of evidence for an outcome.	8
**RESULTS**			
Study selection	16a	Describe the results of the search and selection process, from the number of records identified in the search to the number of studies included in the review, ideally using a flow diagram.	9
	16b	Cite studies that might appear to meet the inclusion criteria, but which were excluded, and explain why they were excluded.	12 (not included into meta-analysis)
Study characteristics	17	Cite each included study and present its characteristics.	10-13
Risk of bias in studies	18	Present assessments of risk of bias for each included study.	13, Table S6
Results of individual studies	19	For all outcomes, present, for each study: (a) summary statistics for each group (where appropriate) and (b) an effect estimate and its precision (e.g. confidence/credible interval), ideally using structured tables or plots.	10-13, Table S2, S3
Results of syntheses	20a	For each synthesis, briefly summarise the characteristics and risk of bias among contributing studies.	10-13
	20b	Present results of all statistical syntheses conducted. If meta-analysis was done, present for each the summary estimate and its precision (e.g. confidence/credible interval) and measures of statistical heterogeneity. If comparing groups, describe the direction of the effect.	10-13

Section and Topic	Item #	Checklist item	Location where item is reported
	20c	Present results of all investigations of possible causes of heterogeneity among study results.	10-13, 14-16
	20d	Present results of all sensitivity analyses conducted to assess the robustness of the synthesized results.	10-11
Reporting biases	21	Present assessments of risk of bias due to missing results (arising from reporting biases) for each synthesis assessed.	10-13
Certainty of evidence	22	Present assessments of certainty (or confidence) in the body of evidence for each outcome assessed.	10-13
**DISCUSSION**			
Discussion	23a	Provide a general interpretation of the results in the context of other evidence.	4,14 (first summary of primary data)
	23b	Discuss any limitations of the evidence included in the review.	17-18
	23c	Discuss any limitations of the review processes used.	17-18
	23d	Discuss implications of the results for practice, policy, and future research.	16
**OTHER INFORMATION**			
Registration and protocol	24a	Provide registration information for the review, including register name and registration number, or state that the review was not registered.	5
	24b	Indicate where the review protocol can be accessed, or state that a protocol was not prepared.	5
	24c	Describe and explain any amendments to information provided at registration or in the protocol.	7
Support	25	Describe sources of financial or non-financial support for the review, and the role of the funders or sponsors in the review.	1
Competing interests	26	Declare any competing interests of review authors.	1
Availability of data, code and other materials	27	Report which of the following are publicly available and where they can be found: template data collection forms; data extracted from included studies; data used for all analyses; analytic code; any other materials used in the review.	1

List S1. Changes made to group counts to avoid multiplicity.

1. Fujihara 2005b, Ferraro 2009 – vector changed from "plasmid+electroporation" to "plasmid"
2. Basu 2014c and Basu 2014d – vector changed from "plasmid+multielectrode array" to "plasmid"
3. Chang 2021a, Chang 2021b, Rah 2014, Lee 2011 – changed flap type from "McFarlane, modified" to "McFarlane"
4. Michlits 2007, O'Toole 2002, Gurunluoglu 2002 – changed flap type from "epigastric, modified" to "epigastric"
5. Hijjawi 2004a,b – split control group count into 3 and 4 (original 7)
6. Rinsch 2001a – changed drug from "VEGF-121" to "VEGF"
7. Taub 1998 - changed drug from "VEGF-121" to "VEGF"
8. O'Toole 2002 - changed drug from "VEGF-167, -186" to "VEGF"
9. Gurunluoglu 2002a – changed drug from "VEGF-164, -166, -167" to "VEGF"
10. Jafari 2017 – control group count split into 2 and 3 from original 5
11. Liu 2009 – control group count split into 3x3 from original 9
12. Giunta 2005 – c and d pooled together (same parameters), control group in a,b,c – 2 each (original 6)
13. Rinsch 2001 a,b – control group count split into 7 and 8 (original 15).
14. Spanholtz 2009 – control group count split into 13,13,14 (original 40)
15. O'Toole 2002 – control group count split into 3,4,4 (original 11)

Figure S1. VEGF comparison by vectors excluding studies at a high risk of bias.

Author & year	VEGF Total	VEGF Mean	VEGF SD	Control Total	Control Mean	Control SD	Administration (day)	Assessment (day)	Mean difference [%]	95% CI
**Virus**										
Antonini 2007a	4	77.6	2.2	4	77.3	2.4	0	7	0.30	[-2.89; 3.49]
Antonini 2007a	4	72.2	1.6	4	63.9	0.6	0	7	8.30	[6.63; 9.97]
Antonini 2007b	4	83.9	3.4	4	74.0	6.0	-7	7	9.90	[3.14; 16.66]
Antonini 2007b	4	76.7	1.6	4	61.1	2.2	-7	7	15.60	[12.93; 18.27]
Antonini 2007c	4	87.7	1.6	4	75.4	1.6	-14	7	12.30	[10.08; 14.52]
Antonini 2007c	4	78.3	2.8	4	62.8	1.4	-14	7	15.50	[12.43; 18.57]
Giunta 2005a	6	29.0	6.4	2	24.6	2.3	0	7	4.35	[-1.65; 10.35]
Giunta 2005b	6	45.2	10.3	2	24.6	2.3	-3	7	20.54	[11.70; 29.38]
Giunta 2005c	12	54.7	5.2	2	24.6	2.3	-7	7	30.01	[25.70; 34.31]
Gurunluoglu 2002a	7	86.0	14.6	7	72.3	34.7	-1	7	13.70	[-14.15; 41.55]
Gurunluoglu 2002b	7	88.7	6.3	7	78.7	18.3	-3	7	10.00	[-4.32; 24.32]
Gurunluoglu 2002c	7	89.3	14.0	7	70.9	36.8	-7	7	18.40	[-10.76; 47.56]
Gurunluoglu 2002d	7	86.9	12.2	7	68.5	26.7	-14	7	18.40	[-3.35; 40.15]
Gurunluoglu 2005	10	88.9	6.1	14	65.6	9.4	0	7	23.30	[17.09; 29.51]
Huang 2006a	6	67.0	2.5	6	54.0	4.9	-7	7	13.00	[8.62; 17.38]
Lubiatowski 2002	12	97.5	1.8	12	82.2	9.7	-2	14	15.40	[9.81; 20.99]
Meirer 2007	10	90.4	3.2	10	82.7	8.2	0	7	7.75	[2.33; 13.17]
Wang 2011	10	55.8	6.9	10	45.9	6.3	-14	7	9.90	[4.11; 15.69]
Zacchinga 2005	20	91.1	2.8	20	77.2	2.7	-14	7	13.90	[12.20; 15.60]
**Random effects model**	**144**			**130**					**15.30**	**[10.16; 20.44]**
Heterogeneity: $I^2 = 86\%$, $\tau^2 = 55.9566$, $p < 0.01$										
**Cell**										
Chen 2011a	16	62.6	8.5	16	36.6	5.3	0	7	26.06	[21.14; 30.98]
Chen 2011b	16	67.0	7.2	16	35.4	4.7	-2	7	31.57	[27.35; 35.79]
Chen 2011c	16	71.4	5.6	16	34.5	4.7	-4	7	36.90	[33.34; 40.46]
Chen 2011d	16	53.7	5.3	16	35.6	4.5	-7	7	18.09	[14.67; 21.51]
Rinsch 2001a	6	47.2	11.3	7	53.2	5.8	0	7	-6.00	[-16.01; 4.01]
Rinsch 2001b	21	46.9	9.7	8	53.2	5.8	0	7	-6.30	[-12.08; -0.52]
Spanholtz 2009a	40	47.6	5.1	13	44.0	3.9	-14	7	3.55	[0.90; 6.20]
Spanholtz 2009b	40	54.0	6.0	13	44.0	3.9	-7	7	9.96	[7.14; 12.78]
Spanholtz 2009c	40	46.5	5.0	14	44.0	3.9	0	7	2.49	[-0.09; 5.07]
Yi 2006	10	68.2	20.4	10	59.4	14.8	-3	7	8.79	[-6.84; 24.42]
Zheng 2008	10	83.1	2.6	10	66.4	6.1	-4	14	16.70	[12.59; 20.81]
**Random effects model**	**231**			**139**					**24.05**	**[16.34; 31.76]**
Heterogeneity: $I^2 = 94\%$, $\tau^2 = 82.3062$, $p < 0.01$										
**Plasmid**										
de Freitas 2010	8	81.7	16.2	8	42.2	25.4	-30	5	39.47	[18.58; 60.36]
Ferraro 2009	7	95.2	5.8	7	70.6	19.6	2	14	24.60	[9.47; 39.73]
Holzbach 2010	6	74.3	15.1	6	49.2	20.4	-7	7	25.10	[4.74; 45.46]
Liu 2004	14	63.7	3.2	14	51.1	3.0	-7	7	12.61	[10.29; 14.93]
Liu1 2005b	10	57.6	5.2	10	50.7	5.9	-7	7	6.90	[2.03; 11.77]
Liu2 2005a	10	57.6	5.2	10	52.3	5.0	-7	7	5.30	[0.83; 9.77]
Michlits 2007	12	86.5	10.7	12	75.4	19.2	0	7	11.13	[-1.30; 23.56]
Neumeister 2001	6	48.8	2.1	6	21.5	2.0	0	7	27.30	[24.98; 29.62]
O'Toole 2002a	14	90.3	8.4	4	83.2	12.1	0	7	7.10	[-5.53; 19.73]
O'Toole 2002b	13	92.1	7.1	4	83.2	12.1	0	7	8.90	[-3.55; 21.35]
O'Toole 2002c	11	86.2	6.6	3	83.2	12.1	0	7	3.00	[-11.21; 17.21]
Rezende 2010	14	50.0	29.5	14	30.6	11.2	0	5	19.35	[2.81; 35.89]
Taub 1998	10	93.9	9.3	10	31.9	9.3	0	7	62.00	[53.83; 70.17]
Yang 2005	10	87.0	5.0	10	47.0	6.0	0	7	40.00	[35.16; 44.84]
Zhang 2005	10	63.7	20.2	10	43.4	11.3	-4	5	20.30	[5.95; 34.65]
**Random effects model**	**155**			**128**					**17.37**	**[10.81; 23.93]**
Heterogeneity: $I^2 = 94\%$, $\tau^2 = 121.1294$, $p < 0.01$										
**Random effects model**	**530**			**397**					**18.03**	**[14.21; 21.86]**
Heterogeneity: $I^2 = 93\%$, $\tau^2 = 93.7743$, $p < 0.01$										
Test for subgroup differences: $\chi^2_2 = 3.45$, df = 2 ($p = 0.18$)										

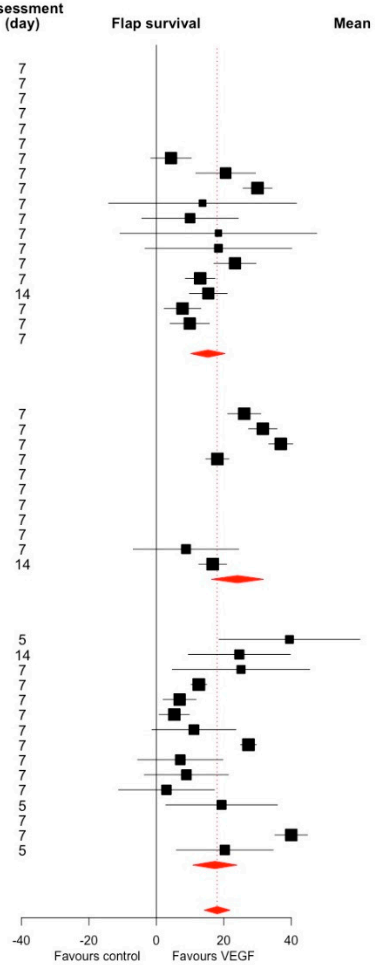

Figure S2. VEGF comparison by vector excluding studies with approximated data extracted from figures.

Author & year	VEGF Total	VEGF Mean	VEGF SD	Control Total	Control Mean	Control SD	Administration (day)	Assessment (day)	Mean difference [%]	95% CI
**Virus**										
Antonini 2007a	4	77.6	2.2	4	77.3	2.4	0	7	0.30	[-2.89; 3.49]
Antonini 2007a	4	72.2	1.6	4	63.9	0.6	0	7	8.30	[6.63; 9.97]
Antonini 2007b	4	83.9	3.4	4	74.0	6.0	-7	7	9.90	[3.14; 16.66]
Antonini 2007b	4	76.7	1.6	4	61.1	2.2	-7	7	15.60	[12.93; 18.27]
Antonini 2007c	4	87.7	1.6	4	75.4	1.6	-14	7	12.30	[10.08; 14.52]
Antonini 2007c	4	78.3	2.8	4	62.8	1.4	-14	7	15.50	[12.43; 18.57]
Giunta 2005a	6	29.0	6.4	2	24.6	2.3	0	7	4.35	[-1.65; 10.35]
Giunta 2005b	6	45.2	10.3	2	24.6	2.3	-3	7	20.54	[11.70; 29.38]
Giunta 2005c	12	54.7	5.2	2	24.6	2.3	-7	7	30.01	[25.70; 34.31]
Gurunluoglu 2002a	7	86.0	14.6	7	72.3	34.7	-1	7	13.70	[-14.15; 41.55]
Gurunluoglu 2002b	7	88.7	6.3	7	78.7	18.3	-3	7	10.00	[-4.32; 24.32]
Gurunluoglu 2002c	7	89.3	14.0	7	70.9	36.8	-7	7	18.40	[-10.76; 47.56]
Gurunluoglu 2002d	7	86.9	12.2	7	68.5	26.7	-14	7	18.40	[-3.35; 40.15]
Gurunluoglu 2005	10	88.9	6.1	14	65.6	9.4	0	7	23.30	[17.09; 29.51]
Huang 2006a	6	67.0	2.5	6	54.0	4.9	-7	7	13.00	[8.62; 17.38]
Lubiatowski 2002	12	97.5	1.8	12	82.2	9.7	-2	14	15.40	[9.81; 20.99]
Meirer 2007	10	90.4	3.2	10	82.7	8.2	0	7	7.75	[2.33; 13.17]
Wang 2011	10	55.8	6.9	10	45.9	6.3	-14	7	9.90	[4.11; 15.69]
Zacchinga 2005	20	91.1	2.8	20	77.2	2.7	-14	7	13.90	[12.20; 15.60]
**Random effects model**	**144**			**130**					**11.92**	**[8.75; 15.10]**
Heterogeneity: $I^2 = 85\%$, $\tau^2 = 26.1212$, $p < 0.01$										
**Cell**										
Chen 2011a	16	62.6	8.5	16	36.6	5.3	0	7	26.06	[21.14; 30.98]
Chen 2011b	16	67.0	7.2	16	35.4	4.7	-2	7	31.57	[27.35; 35.79]
Chen 2011c	16	71.4	5.6	16	34.5	4.7	-4	7	36.90	[33.34; 40.46]
Chen 2011d	16	53.7	5.3	16	35.6	4.5	-7	7	18.09	[14.67; 21.51]
Rinsch 2001a	6	47.2	11.3	7	53.2	5.8	0	7	-6.00	[-16.01; 4.01]
Rinsch 2001b	21	46.9	9.7	8	53.2	5.8	0	7	-6.30	[-12.08; -0.52]
Spanholtz 2009a	40	47.6	5.1	13	44.0	3.9	-14	7	3.55	[0.90; 6.20]
Spanholtz 2009b	40	54.0	6.0	13	44.0	3.9	-7	7	9.96	[7.14; 12.78]
Spanholtz 2009c	40	46.5	5.0	14	44.0	3.9	0	7	2.49	[-0.09; 5.07]
Yi 2006	10	68.2	20.4	10	59.4	14.8	-3	7	8.79	[-6.84; 24.42]
Zheng 2008	10	83.1	2.6	10	66.4	6.1	-4	14	16.70	[12.59; 20.81]
**Random effects model**	**231**			**139**					**17.00**	**[4.47; 29.54]**
Heterogeneity: $I^2 = 97\%$, $\tau^2 = 278.4724$, $p < 0.01$										
**Plasmid**										
de Freitas 2010	8	81.7	16.2	8	42.2	25.4	-30	5	39.47	[18.58; 60.36]
Ferraro 2009	7	95.2	5.8	7	70.6	19.6	2	14	24.60	[9.47; 39.73]
Holzbach 2010	6	74.3	15.1	6	49.2	20.4	-7	7	25.10	[4.74; 45.46]
Liu 2004	14	63.7	3.2	14	51.1	3.0	-7	7	12.61	[10.29; 14.93]
Liu1 2005b	10	57.6	5.2	10	50.7	5.9	-7	7	6.90	[2.03; 11.77]
Liu2 2005a	10	57.6	5.2	10	52.3	5.0	-7	7	5.30	[0.83; 9.77]
Michlits 2007	12	86.5	10.7	12	75.4	19.2	0	7	11.13	[-1.30; 23.56]
Neumeister 2001	6	48.8	2.1	6	21.5	2.0	0	7	27.30	[24.98; 29.62]
O'Toole 2002a	14	90.3	8.4	4	83.2	12.1	0	7	7.10	[-5.53; 19.73]
O'Toole 2002b	13	92.1	7.1	4	83.2	12.1	0	7	8.90	[-3.55; 21.35]
O'Toole 2002c	11	86.2	6.6	3	83.2	12.1	0	7	3.00	[-11.21; 17.21]
Rezende 2010	14	50.0	29.5	14	30.6	11.2	0	5	19.35	[2.81; 35.89]
Taub 1998	10	93.9	9.3	10	31.9	9.3	0	7	62.00	[53.83; 70.17]
Yang 2005	10	87.0	5.0	10	47.0	6.0	0	7	40.00	[35.16; 44.84]
Zhang 2005	10	63.7	20.2	10	43.4	11.3	-4	5	20.30	[5.95; 34.65]
**Random effects model**	**155**			**128**					**22.53**	**[11.81; 33.25]**
Heterogeneity: $I^2 = 96\%$, $\tau^2 = 180.6110$, $p < 0.01$										
**Random effects model**	**530**			**397**					**15.94**	**[11.52; 20.37]**
Heterogeneity: $I^2 = 96\%$, $\tau^2 = 126.7628$, $p < 0.01$										
Test for subgroup differences: $\chi^2_2 = 3.87$, df = 2 ($p = 0.14$)										

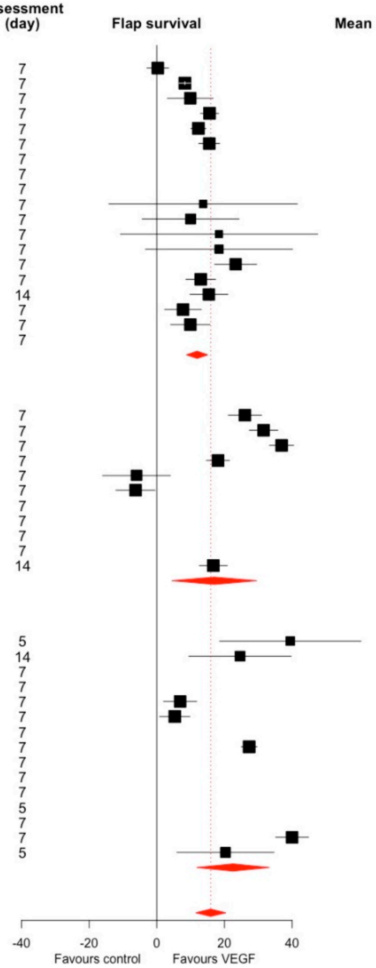

Figure S3. VEGF comparison using both random effects and fixed effect models.

Author & year	VEGF Total	VEGF Mean	VEGF SD	Control Total	Control Mean	Control SD	Administration (day)	Assessment (day)	Mean difference [%]	95% CI
**Virus**										
Antonini 2007a	4	77.6	2.2	4	77.3	2.4	0	7	0.30	[-2.89; 3.49]
Antonini 2007a	4	72.2	1.6	4	63.9	0.6	0	7	8.30	[6.63; 9.97]
Antonini 2007b	4	83.9	3.4	4	74.0	6.0	-7	7	9.90	[3.14; 16.66]
Antonini 2007b	4	76.7	1.6	4	61.1	2.2	-7	7	15.60	[12.93; 18.27]
Antonini 2007c	4	87.7	1.6	4	75.4	1.6	-14	7	12.30	[10.08; 14.52]
Antonini 2007c	4	78.3	2.8	4	62.8	1.4	-14	7	15.50	[12.43; 18.57]
Giunta 2005a	6	29.0	6.4	2	24.6	2.3	0	7	4.35	[-1.65; 10.35]
Giunta 2005b	6	45.2	10.3	2	24.6	2.3	-3	7	20.54	[11.70; 29.38]
Giunta 2005c	12	54.7	5.2	2	24.6	2.3	-7	7	30.01	[25.70; 34.31]
Gurunluoglu 2002a	7	86.0	14.6	7	72.3	34.7	-1	7	13.70	[-14.15; 41.55]
Gurunluoglu 2002b	7	88.7	6.3	7	78.7	18.3	-3	7	10.00	[-4.32; 24.32]
Gurunluoglu 2002c	7	89.3	14.0	7	70.9	36.8	-7	7	18.40	[-10.76; 47.56]
Gurunluoglu 2002d	7	86.9	12.2	7	68.5	26.7	-14	7	18.40	[-3.35; 40.15]
Gurunluoglu 2005	10	88.9	6.1	14	65.6	9.4	0	7	23.30	[17.09; 29.51]
Huang 2006a	6	67.0	2.5	6	54.0	4.9	-7	7	13.00	[8.62; 17.38]
Lubiatowski 2002	12	97.5	1.8	12	82.2	9.7	-2	14	15.40	[9.81; 20.99]
Meirer 2007	10	90.4	3.2	10	82.7	8.2	0	7	7.75	[2.33; 13.17]
Wang 2011	10	55.8	6.9	10	45.9	6.3	-14	7	9.90	[4.11; 15.69]
Zacchinga 2005	20	91.1	2.8	20	77.2	2.7	-14	7	13.90	[12.20; 15.60]
**Common effect model**	**144**			**130**					**12.14**	**[11.33; 12.94]**
**Random effects model**									**13.27**	**[9.80; 16.74]**
Heterogeneity: $I^2 = 90\%$, $\tau^2 = 44.8875$, $p < 0.01$										
**Cell**										
Chen 2011a	16	62.6	8.5	16	36.6	5.3	0	7	26.06	[21.14; 30.98]
Chen 2011b	16	67.0	7.2	16	35.4	4.7	-2	7	31.57	[27.35; 35.79]
Chen 2011c	16	71.4	5.6	16	34.5	4.7	-4	7	36.90	[33.34; 40.46]
Chen 2011d	16	53.7	5.3	16	35.6	4.5	-7	7	18.09	[14.67; 21.51]
Rinsch 2001a	6	47.2	11.3	7	53.2	5.8	0	7	-6.00	[-16.01; 4.01]
Rinsch 2001b	21	46.9	9.7	8	53.2	5.8	0	7	-6.30	[-12.08; -0.52]
Spanholtz 2009a	40	47.6	5.1	13	44.0	3.9	-14	7	3.55	[0.90; 6.20]
Spanholtz 2009b	40	54.0	6.0	13	44.0	3.9	-7	7	9.96	[7.14; 12.78]
Spanholtz 2009c	40	46.5	5.0	14	44.0	3.9	0	7	2.49	[-0.09; 5.07]
Yi 2006	10	68.2	20.4	10	59.4	14.8	-3	7	8.79	[-6.84; 24.42]
Zheng 2008	10	83.1	2.6	10	66.4	6.1	-4	14	16.70	[12.59; 20.81]
**Common effect model**	**231**			**139**					**13.04**	**[11.91; 14.17]**
**Random effects model**									**13.16**	**[4.55; 21.76]**
Heterogeneity: $I^2 = 98\%$, $\tau^2 = 201.9151$, $p < 0.01$										
**Plasmid**										
de Freitas 2010	8	81.7	16.2	8	42.2	25.4	-30	5	39.47	[18.58; 60.36]
Ferraro 2009	7	95.2	5.8	7	70.6	19.6	2	14	24.60	[9.47; 39.73]
Holzbach 2010	6	74.3	15.1	6	49.2	20.4	-7	7	25.10	[4.74; 45.46]
Liu 2004	14	63.7	3.2	14	51.1	3.0	-7	7	12.61	[10.29; 14.93]
Liu1 2005b	10	57.6	5.2	10	50.7	5.9	-7	7	6.90	[2.03; 11.77]
Liu2 2005a	10	57.6	5.2	10	52.3	5.0	-7	7	5.30	[0.83; 9.77]
Michlits 2007	12	86.5	10.7	12	75.4	19.2	0	7	11.13	[-1.30; 23.56]
Neumeister 2001	6	48.8	2.1	6	21.5	2.0	0	7	27.30	[24.98; 29.62]
O'Toole 2002a	14	90.3	8.4	4	83.2	12.1	0	7	7.10	[-5.53; 19.73]
O'Toole 2002b	13	92.1	7.1	4	83.2	12.1	0	7	8.90	[-3.55; 21.35]
O'Toole 2002c	11	86.2	6.6	3	83.2	12.1	0	7	3.00	[-11.21; 17.21]
Rezende 2010	14	50.0	29.5	14	30.6	11.2	0	5	19.35	[2.81; 35.89]
Taub 1998	10	93.9	9.3	10	31.9	9.3	0	7	62.00	[53.83; 70.17]
Yang 2005	10	87.0	5.0	10	47.0	6.0	0	7	40.00	[35.16; 44.84]
Zhang 2005	10	63.7	20.2	10	43.4	11.3	-4	5	20.30	[5.95; 34.65]
**Common effect model**	**155**			**128**					**19.92**	**[18.59; 21.25]**
**Random effects model**									**20.74**	**[12.13; 29.35]**
Heterogeneity: $I^2 = 96\%$, $\tau^2 = 252.3387$, $p < 0.01$										
**Common effect model**	**530**			**397**					**13.89**	**[13.31; 14.48]**
**Random effects model**									**15.66**	**[11.80; 19.52]**

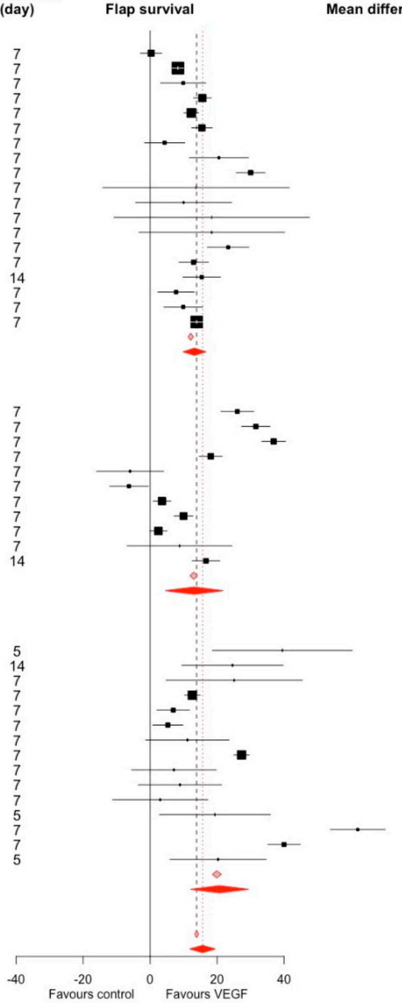

Heterogeneity: $I^2 = 96\%$, $\tau^2 = 151.3318$, $p < 0.01$
Test for subgroup differences (common effect): $\chi^2_2 = 99.25$, df = 2 ($p < 0.01$)
Test for subgroup differences (random effects): $\chi^2_2 = 2.55$, df = 2 ($p = 0.28$)

Figure S4. VEGF comparison by flap excluding studies with approximated data extracted from figures.

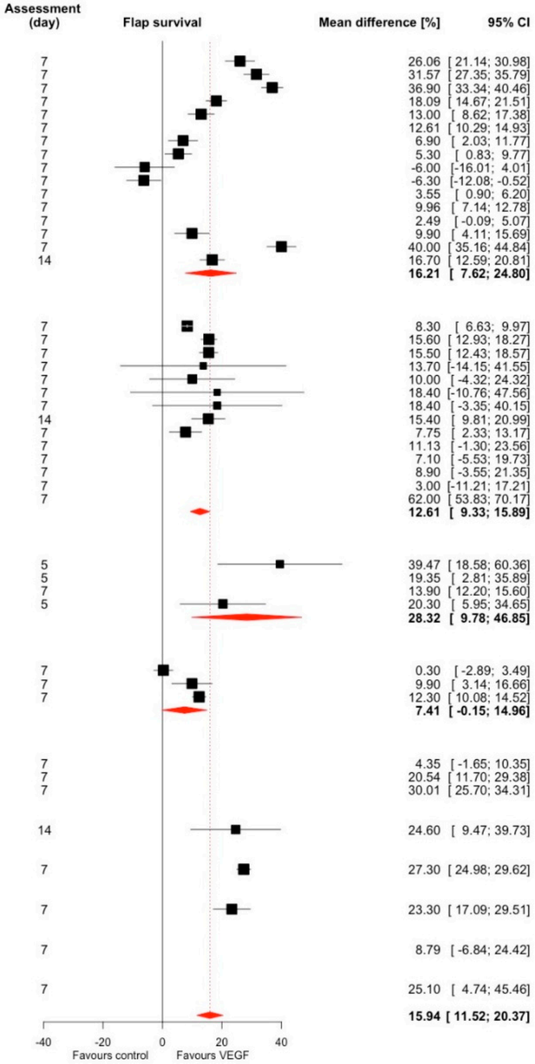

Author & year	VEGF Total	VEGF Mean	VEGF SD	Control Total	Control Mean	Control SD	Administration (day)	Assessment (day)	Mean difference [%]	95% CI
**McFarlane**										
Chen 2011a	16	62.6	8.5	16	36.6	5.3	0	7	26.06	[21.14; 30.98]
Chen 2011b	16	67.0	7.2	16	35.4	4.7	-2	7	31.57	[27.35; 35.79]
Chen 2011c	16	71.4	5.6	16	34.5	4.7	-4	7	36.90	[33.34; 40.46]
Chen 2011d	16	53.7	5.3	16	35.6	4.5	-7	7	18.09	[14.67; 21.51]
Huang 2006a	6	67.0	2.5	6	54.0	4.9	-7	7	13.00	[8.62; 17.38]
Liu 2004	14	63.7	3.2	14	51.1	3.0	-7	7	12.61	[10.29; 14.93]
Liu1 2005b	10	57.6	5.2	10	50.7	5.9	-7	7	6.90	[2.03; 11.77]
Liu2 2005a	10	57.6	5.2	10	52.3	5.0	-7	7	5.30	[0.83; 9.77]
Rinsch 2001a	6	47.2	11.3	7	53.2	5.8	0	7	-6.00	[-16.01; 4.01]
Rinsch 2001b	21	46.9	9.7	8	53.2	5.8	0	7	-6.30	[-12.08; -0.52]
Spanholtz 2009a	40	47.6	5.1	13	44.0	3.9	-14	7	3.55	[0.90; 6.20]
Spanholtz 2009b	40	54.0	6.0	13	44.0	3.9	-7	7	9.96	[7.14; 12.78]
Spanholtz 2009c	40	46.5	5.0	14	44.0	3.9	0	7	2.49	[-0.09; 5.07]
Wang 2011	10	55.8	6.9	10	45.9	6.3	-14	7	9.90	[4.11; 15.69]
Yang 2005	10	87.0	5.0	10	47.0	6.0	0	7	40.00	[35.16; 44.84]
Zheng 2008	10	83.1	2.6	10	66.4	6.1	-4	14	16.70	[12.59; 20.81]
**Random effects model**	**281**			**189**					**16.21**	**[7.62; 24.80]**
Heterogeneity: $I^2 = 97\%$, $\tau^2 = 223.4412$, $p < 0.01$										
**Epigastric**										
Antonini 2007a	4	72.2	1.6	4	63.9	0.6	0	7	8.30	[6.63; 9.97]
Antonini 2007b	4	76.7	1.6	4	61.1	2.2	-7	7	15.60	[12.93; 18.27]
Antonini 2007c	4	78.3	2.8	4	62.8	1.4	-14	7	15.50	[12.43; 18.57]
Gurunluoglu 2002a	7	86.0	14.6	7	72.3	34.7	-1	7	13.70	[-14.15; 41.55]
Gurunluoglu 2002b	7	88.7	6.3	7	78.7	18.3	-3	7	10.00	[-4.32; 24.32]
Gurunluoglu 2002c	7	89.3	14.0	7	70.9	36.8	-7	7	18.40	[-10.76; 47.56]
Gurunluoglu 2002d	7	86.9	12.2	7	68.5	26.7	-14	7	18.40	[-3.35; 40.15]
Lubiatowski 2002	12	97.5	1.8	12	82.2	9.7	-2	14	15.40	[9.81; 20.99]
Meirer 2007	10	90.4	3.2	10	82.7	8.2	0	7	7.75	[2.33; 13.17]
Michlits 2007	12	86.5	10.7	12	75.4	19.2	0	7	11.13	[-1.30; 23.56]
O'Toole 2002a	14	90.3	8.4	4	83.2	12.1	0	7	7.10	[-5.53; 19.73]
O'Toole 2002b	13	92.1	7.1	4	83.2	12.1	0	7	8.90	[-3.55; 21.35]
O'Toole 2002c	11	86.2	6.6	3	83.2	12.1	0	7	3.00	[-11.21; 17.21]
Taub 1998	10	93.9	9.3	10	31.9	9.3	0	7	62.00	[53.83; 70.17]
**Random effects model**	**122**			**95**					**12.61**	**[9.33; 15.89]**
Heterogeneity: $I^2 = 76\%$, $\tau^2 = 11.7605$, $p < 0.01$										
**TRAM**										
de Freitas 2010	8	81.7	16.2	8	42.2	25.4	-30	5	39.47	[18.58; 60.36]
Rezende 2010	14	50.0	29.5	14	30.6	11.2	0	5	19.35	[2.81; 35.89]
Zacchinga 2005	20	91.1	2.8	20	77.2	2.7	-14	7	13.90	[12.20; 15.60]
Zhang 2005	10	63.7	20.2	10	43.4	11.3	-4	5	20.30	[5.95; 34.65]
**Random effects model**	**52**			**52**					**28.32**	**[9.78; 46.85]**
Heterogeneity: $I^2 = 54\%$, $\tau^2 = 100.1376$, $p = 0.14$										
**Musculocutaneous**										
Antonini 2007a	4	77.6	2.2	4	77.3	2.4	0	7	0.30	[-2.89; 3.49]
Antonini 2007b	4	83.9	3.4	4	74.0	6.0	-7	7	9.90	[3.14; 16.66]
Antonini 2007c	4	87.7	1.6	4	75.4	1.6	-14	7	12.30	[10.08; 14.52]
**Random effects model**	**12**			**12**					**7.41**	**[-0.15; 14.96]**
Heterogeneity: $I^2 = 95\%$, $\tau^2 = 39.7334$, $p < 0.01$										
**Abdominal random-pattern flap**										
Giunta 2005a	6	29.0	6.4	2	24.6	2.3	0	7	4.35	[-1.65; 10.35]
Giunta 2005b	6	45.2	10.3	2	24.6	2.3	-3	7	20.54	[11.70; 29.38]
Giunta 2005c	12	54.7	5.2	2	24.6	2.3	-7	7	30.01	[25.70; 34.31]
**Single pedicle random skin flap**										
Ferraro 2009	7	95.2	5.8	7	70.6	19.6	2	14	24.60	[9.47; 39.73]
**Gracilis**										
Neumeister 2001	6	48.8	2.1	6	21.5	2.0	0	7	27.30	[24.98; 29.62]
**Peninsular abdominal flap**										
Gurunluoglu 2005	10	88.9	6.1	14	65.6	9.4	0	7	23.30	[17.09; 29.51]
**Cranially based flap**										
Yi 2006	10	68.2	20.4	10	59.4	14.8	-3	7	8.79	[-6.84; 24.42]
**Skin flap with pedicle**										
Holzbach 2010	6	74.3	15.1	6	49.2	20.4	-7	7	25.10	[4.74; 45.46]
**Random effects model**	**530**			**397**					**15.94**	**[11.52; 20.37]**
Heterogeneity: $I^2 = 96\%$, $\tau^2 = 126.7628$, $p < 0.01$										
Test for subgroup differences: $\chi^2_6 = 68.07$, df = 6 ($p < 0.01$)										

Figure S5. VEGF comparison by flap type with both random effects and fixed effect model.

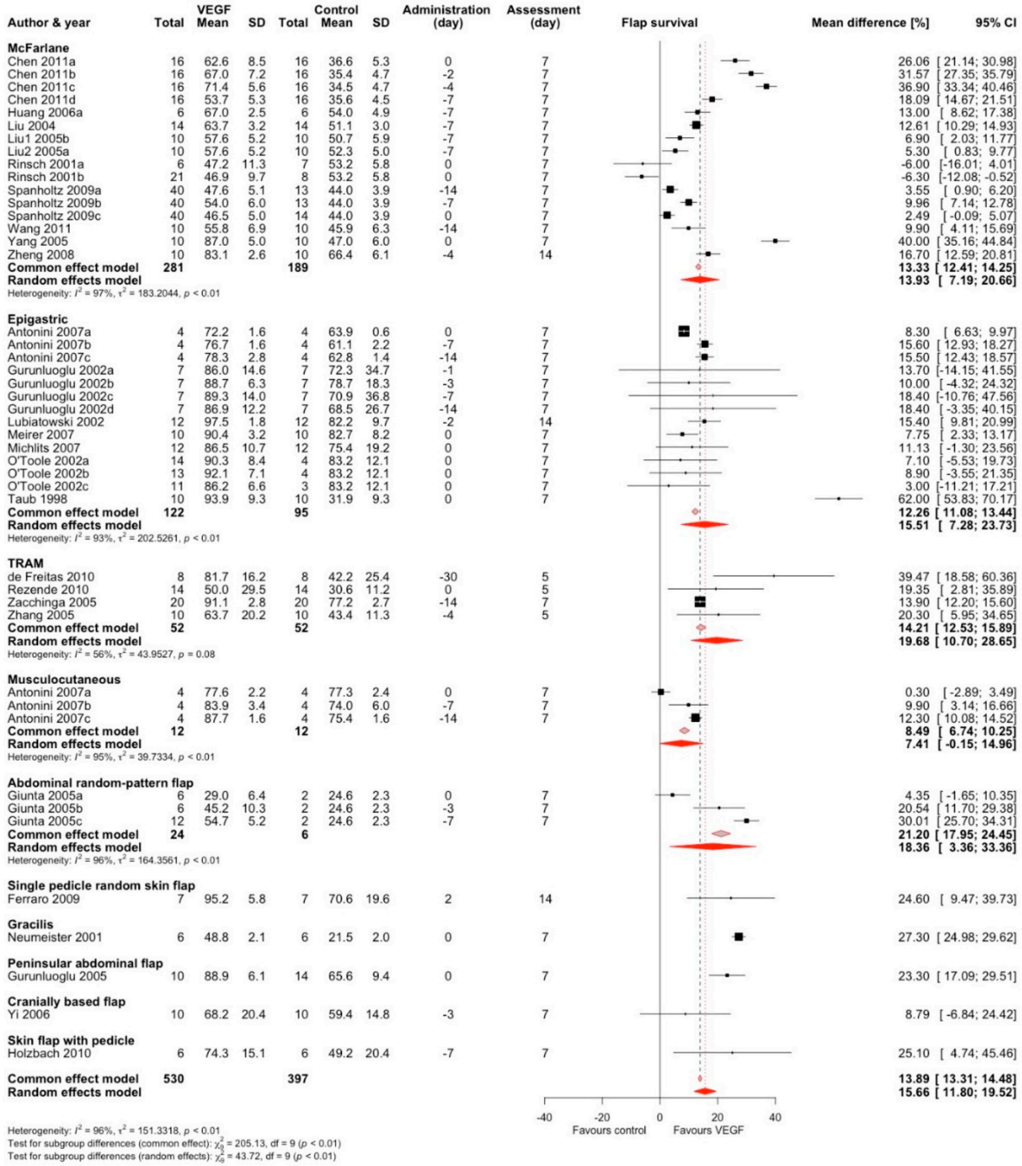

Figure S6. VEGF comparison by administration route.

Author & year	VEGF Total	VEGF Mean	VEGF SD	Control Total	Control Mean	Control SD	Administration (day)	Assessment (day)	Mean difference [%]	95% CI
**Intramuscular**										
Antonini 2007a	4	77.6	2.2	4	77.3	2.4	0	7	0.30	[-2.89; 3.49]
Antonini 2007b	4	83.9	3.4	4	74.0	6.0	-7	7	9.90	[3.14; 16.66]
Antonini 2007c	4	87.7	1.6	4	75.4	1.6	-14	7	12.30	[10.08; 14.52]
Neumeister 2001	6	48.8	2.1	6	21.5	2.0	0	7	27.30	[24.98; 29.62]
Zacchinga 2005	20	91.1	2.8	20	77.2	2.7	-14	7	13.90	[12.20; 15.60]
**Random effects model**	**38**			**38**					**12.83**	**[4.21; 21.45]**
Heterogeneity: $I^2 = 98\%$, $\tau^2 = 93.2979$, $p < 0.01$										
**Subcutaneous**										
Antonini 2007a	4	72.2	1.6	4	63.9	0.6	0	7	8.30	[6.63; 9.97]
Antonini 2007b	4	76.7	1.6	4	61.1	2.2	-7	7	15.60	[12.93; 18.27]
Antonini 2007c	4	78.3	2.8	4	62.8	1.4	-14	7	15.50	[12.43; 18.57]
Chen 2011a	16	62.6	8.5	16	36.6	5.3	0	7	26.06	[21.14; 30.98]
Chen 2011b	16	67.0	7.2	16	35.4	4.7	-2	7	31.57	[27.35; 35.79]
Chen 2011c	16	71.4	5.6	16	34.5	4.7	-4	7	36.90	[33.34; 40.46]
Chen 2011d	16	53.7	5.3	16	35.6	4.5	-7	7	18.09	[14.67; 21.51]
Giunta 2005a	6	29.0	6.4	2	24.6	2.3	0	7	4.35	[-1.65; 10.35]
Giunta 2005b	6	45.2	10.3	2	24.6	2.3	-3	7	20.54	[11.70; 29.38]
Giunta 2005c	12	54.7	5.2	2	24.6	2.3	-7	7	30.01	[25.70; 34.31]
Gurunluoglu 2002a	7	86.0	14.6	7	72.3	34.7	-1	7	13.70	[-14.15; 41.55]
Gurunluoglu 2002b	7	88.7	6.3	7	78.7	18.3	-3	7	10.00	[-4.32; 24.32]
Gurunluoglu 2002c	7	89.3	14.0	7	70.9	36.8	-7	7	18.40	[-10.76; 47.56]
Gurunluoglu 2002d	7	86.9	12.2	7	68.5	26.7	-14	7	18.40	[-3.35; 40.15]
Gurunluoglu 2005	10	88.9	6.1	14	65.6	9.4	0	7	23.30	[17.09; 29.51]
Holzbach 2010	6	74.3	15.1	6	49.2	20.4	-7	7	25.10	[4.74; 45.46]
Huang 2006a	6	67.0	2.5	6	54.0	4.9	-7	7	13.00	[8.62; 17.38]
Lubiatowski 2002	12	97.5	1.8	12	82.2	9.7	-2	14	15.40	[9.81; 20.99]
Meirer 2007	10	90.4	3.2	10	82.7	8.2	0	7	7.75	[2.33; 13.17]
O'Toole 2002a	14	90.3	8.4	4	83.2	12.1	0	7	7.10	[-5.53; 19.73]
O'Toole 2002b	13	92.1	7.1	4	83.2	12.1	0	7	8.90	[-3.55; 21.35]
O'Toole 2002c	11	86.2	6.6	3	83.2	12.1	0	7	3.00	[-11.21; 17.21]
Rinsch 2001a	6	47.2	11.3	7	53.2	5.8	0	7	-6.00	[-16.01; 4.01]
Rinsch 2001b	21	46.9	9.7	8	53.2	5.8	0	7	-6.30	[-12.08; -0.52]
Yang 2005	10	87.0	5.0	10	47.0	6.0	0	7	40.00	[35.16; 44.84]
Yi 2006	10	68.2	20.4	10	59.4	14.8	-3	7	8.79	[-6.84; 24.42]
Zhang 2005	10	63.7	20.2	10	43.4	11.3	-4	5	20.30	[5.95; 34.65]
Zheng 2008	10	83.1	2.6	10	66.4	6.1	-4	14	16.70	[12.59; 20.81]
**Random effects model**	**277**			**224**					**16.18**	**[11.64; 20.71]**
Heterogeneity: $I^2 = 95\%$, $\tau^2 = 123.4743$, $p < 0.01$										
**Intrafascial**										
de Freitas 2010	8	81.7	16.2	8	42.2	25.4	-30	5	39.47	[18.58; 60.36]
**Intradermal**										
Ferraro 2009	7	95.2	5.8	7	70.6	19.6	2	14	24.60	[9.47; 39.73]
Liu 2004	14	63.7	3.2	14	51.1	3.0	-7	7	12.61	[10.29; 14.93]
Liu1 2005b	10	57.6	5.2	10	50.7	5.9	-7	7	6.90	[2.03; 11.77]
Liu2 2005a	10	57.6	5.2	10	52.3	5.0	-7	7	5.30	[0.83; 9.77]
Rezende 2010	14	50.0	29.5	14	30.6	11.2	0	5	19.35	[2.81; 35.89]
Spanholtz 2009a	40	47.6	5.1	13	44.0	3.9	-14	7	3.55	[0.90; 6.20]
Spanholtz 2009b	40	54.0	6.0	13	44.0	3.9	-7	7	9.96	[7.14; 12.78]
Spanholtz 2009c	40	46.5	5.0	14	44.0	3.9	0	7	2.49	[-0.09; 5.07]
Wang 2011	10	55.8	6.9	10	45.9	6.3	-14	7	9.90	[4.11; 15.69]
**Random effects model**	**185**			**105**					**8.17**	**[4.99; 11.34]**
Heterogeneity: $I^2 = 85\%$, $\tau^2 = 16.0959$, $p < 0.01$										
**Topical/fibrin sealant**										
Michlits 2007	12	86.5	10.7	12	75.4	19.2	0	7	11.13	[-1.30; 23.56]
**Intraarterial**										
Taub 1998	10	93.9	9.3	10	31.9	9.3	0	7	62.00	[53.83; 70.17]
**Random effects model**	**530**			**397**					**15.66**	**[11.80; 19.52]**
Heterogeneity: $I^2 = 96\%$, $\tau^2 = 151.3318$, $p < 0.01$										
Test for subgroup differences: $\chi^2_5 = 150.79$, df = 5 ($p < 0.01$)										

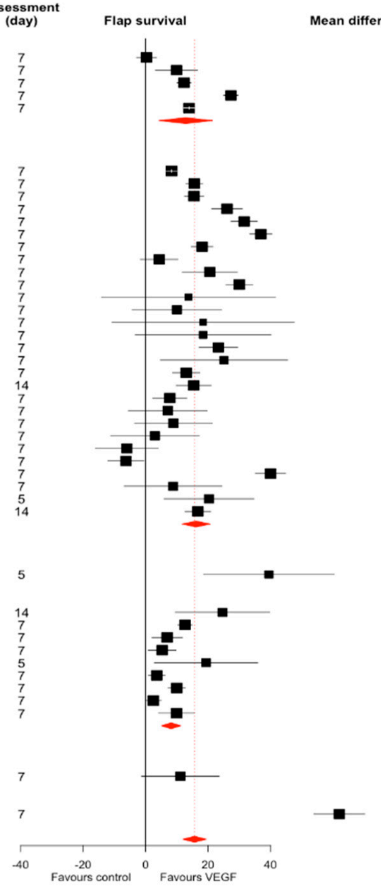

Table S2. Multivariate meta-regression model for VEGF intervention.

Variable	Effect estimate (95% confidence interval)	p-value
Assessment time	0.61 (-1.19 to 2.42)	0.51
Administration time	-0.42 (-1.21 to 0.37)	0.29
Plasmid (vector) [a]	6.19 (-3.13 to 15.52)	0.19
Virus (vector) [a]	-2.32 (-10.99 to 6.34)	0.60
Intramuscular (RoD) [b]	6.90 (-5.57 to 19.37)	0.28
**Subcutaneous (RoD) [b]**	**11.00 (1.47 to 20.53)**	**0.024**

RoD – route of administration; [a] – reference: cell (vector); [b] – reference: intradermal (RoD)

Figure S7. Funnel plot regarding publication bias showing VEGF intervention group.

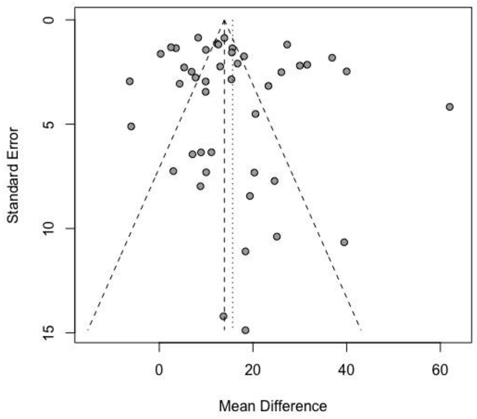

<u>VEGF</u>

intercept 95% CI t p

1.192 -1.33 - 3.72 0.926 0.3597875

Eggers' test does not indicate the presence of funnel plot asymmetry.

Figure S8. FGF comparison by vector excluding studies at a high risk of bias.

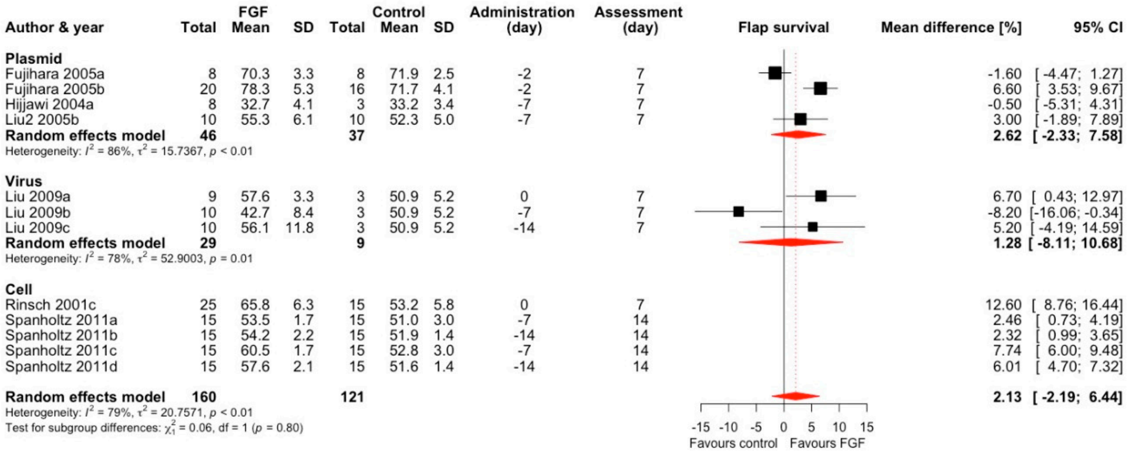

Figure S9. FGF comparison by vector excluding studies with data approximated from figures.

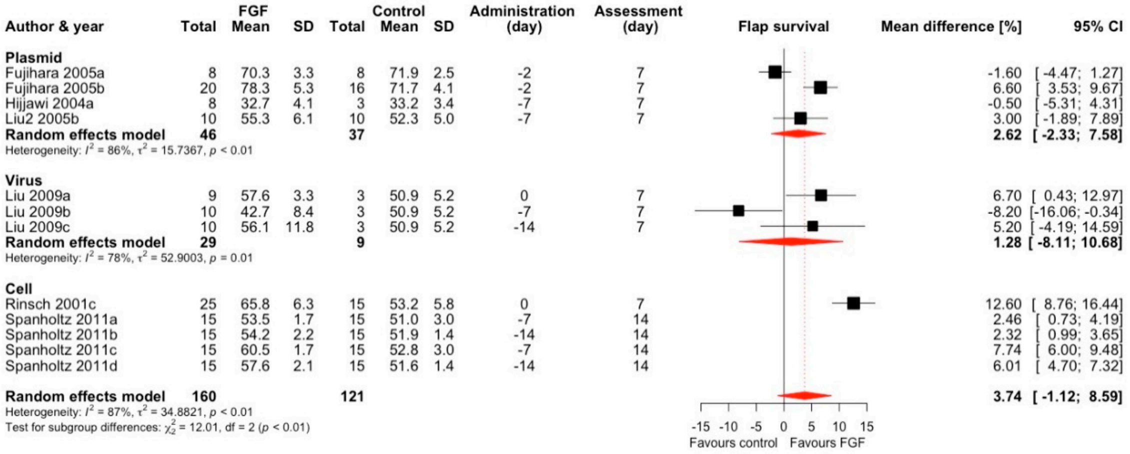

Figure S10. Funnel plot regarding publication bias showing FGF intervention group.

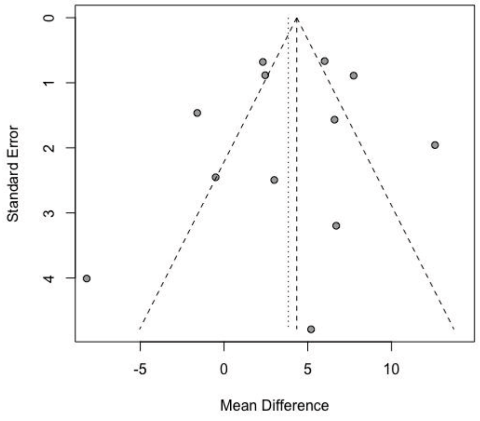

<u>FGF</u>

intercept	95% CI	t	p
-0.578	-3.68 - 2.53	-0.365	0.7230062

Eggers' test does not indicate the presence of funnel plot asymmetry.

Figure S11. PDGF comparison by vector excluding studies at a high risk of bias.

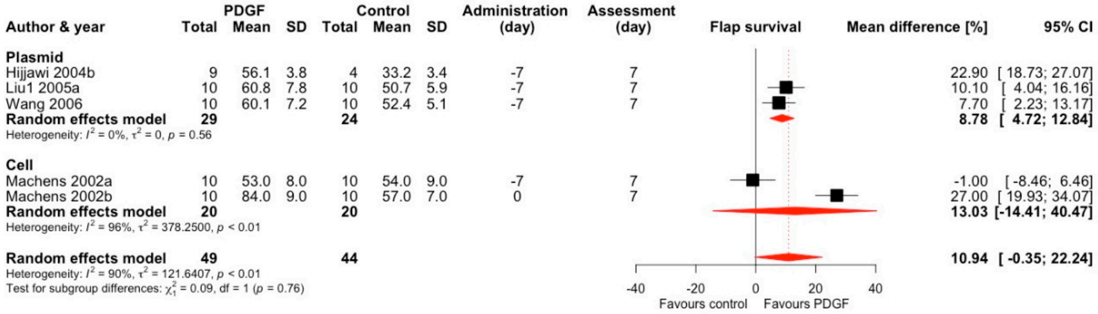

Figure S12. PDGF comparison by vector excluding studies with data approximated from figures.

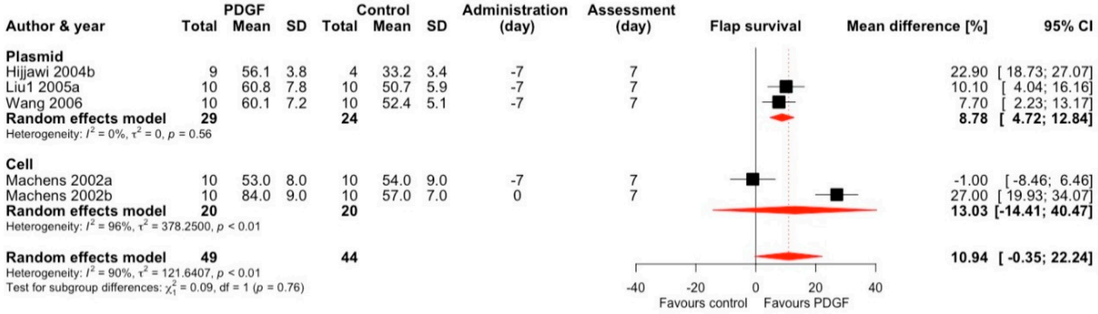

Figure S13. PDGF comparison by vector using both random effects and fixed effect models.

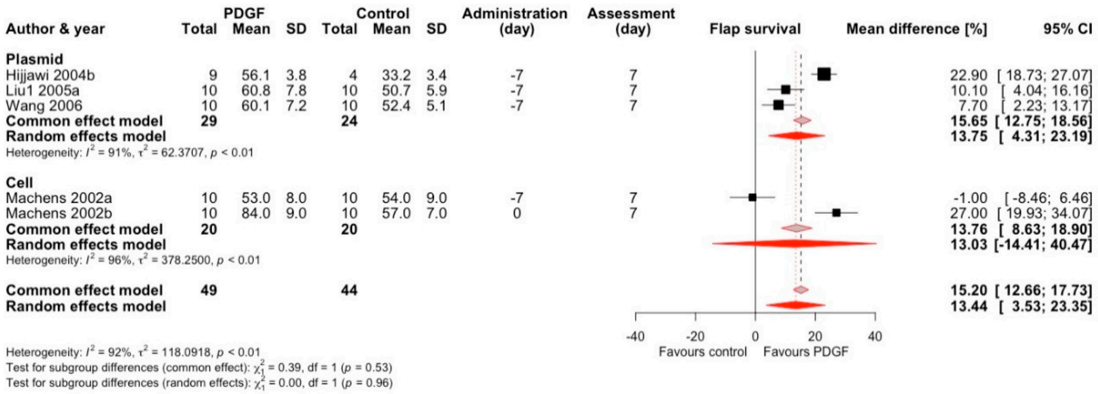

Figure S14. PDGF comparison by flap excluding studies at a high risk of bias.

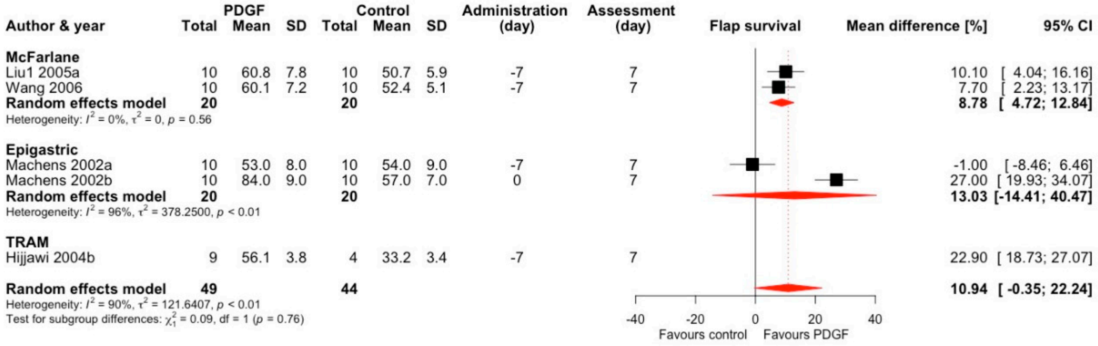

Figure S15. PDGF comparison by flap type excluding studies with data approximated from figures.

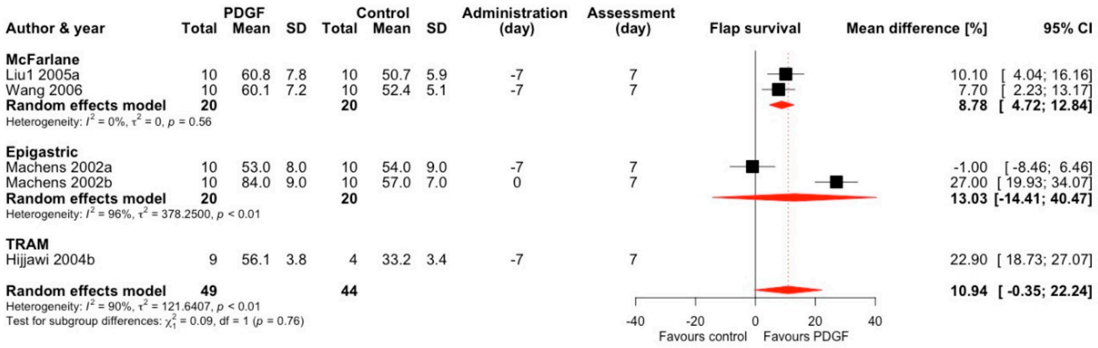

Figure S16. PDGF comparison by flap using both random effects and fixed effect models.

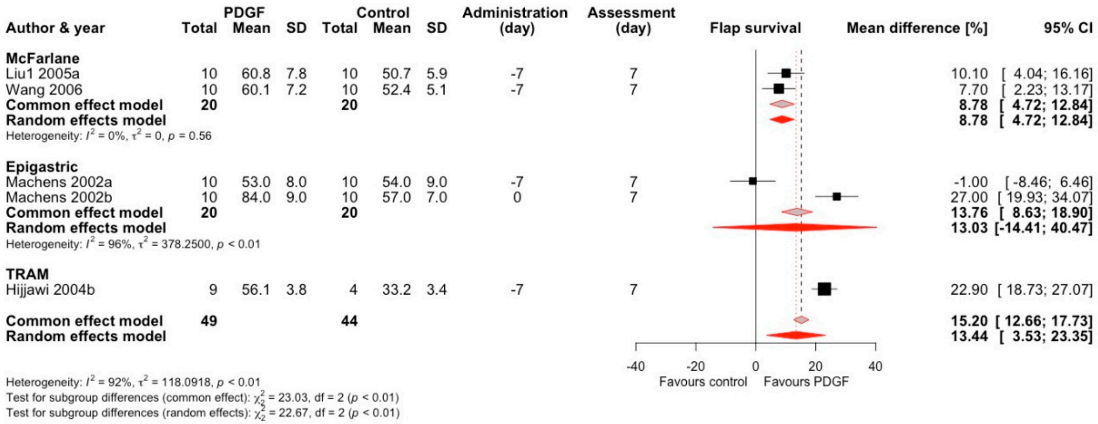

Table S3. Overview of studies included in secondary outcome analysis.

1st Author; Year	Target gene	Focus	Vector amount	Experimental groups	Outcome	Follow-up	Flap model	Animal number	Surgical technique	Secondary outcomes (with P values)
Neumeister; 2001	VEGF-165	Increasing viability of flaps	50 µl	(A) lipofectamine and VEGF plasmid (n=6),<br>(B) lipofectamine (n=6)	Capillary-to-muscle fiber ratio	7 days	gracilis muscle microcirculation model	12 Wistar male rats (300-325g)	Intramuscular injection at the end of 4-hours of ischemia induced by microvascular clamp of main femoral vessels	(A) 1.16±0.09<br>(B) 0.48±0.07<br>P<0.05 (A) vs. (B)
Liu; 2005	PDGF-B VEGF	Increasing survival and vascularity of the ischemic flap	50 mg	(A) PDGF plasmid (n=10)<br>(B) saline (n=10),<br>(C) empty plasmid (n=10)<br>(D) VEGF plasmid (n=10)	Flap vascularity (blood vessel density)	7 days	Caudally-based random pattern McFarlane flap, 3x10cm	45 Sprague-Dawley female rats (250-300g)	Intradermal injection (7 days before flap elevation).	The density of blood vessels:<br>(A) 6.8±3.4<br>(C) 3.8±2.6<br>P<0.05
Liu; 2004	VEGF-165	Increasing flap survival and angiogenesis around flap	50 mg	(A) VEGF plasmid (n=14)<br>(B) saline (n=4)<br>(C) Lac-Z plasmid (n=14)	Neovascularization	7 days	Random pattern McFarlane flap, 3x10cm	32 Sprague-Dawley female rats (250g)	Intradermal injection (7 days prior to flap elevation).	Blood vessel counts (n/hpf)<br>(C) – 6.0<br>(A) - 13.9<br>P<0.001
Liu; 2005	VEGF-165 PDGF-B bFGF	Enhancement of survival of ischemic skin flaps	50 µg	(A) VEGF165 plasmid (n=10)<br>(B) bFGF plasmid (n=10)<br>(C) VEGF, bFGF plasmid (n=10)<br>(D) VEGF165, PDGF-B plasmid (n=10)<br>(E) VEGF165, bFGF, PDGF-B plasmid (n=10)<br>(F) Empty plasmid (n=10)	Neovascularization<br><br>Transfection efficiency	7 days	Random pattern McFarlane flap, 3x10cm	60 Sprague-Dawley female rats (250-300g)	Intradermal injection (7 days prior to flap elevation)	Blood vessel counts (n/hpf)<br>(A) - 13.6±3.2<br>(B) – 7.2±2.7<br>(C) – 12.0±4.0<br>(D) – 14.0±5.0<br>(E) – 35.1±10.8<br>(F) – 6.3±2.3<br>All P<0.05
Huang; 2006	VEGF-165 eNOS	Increasing skin flap viability, increasing synthesis/release of the angiogenic and vasodilator factors	VEGF: 5 x 10^8 PFU<br>eNOS: 5 x 10^8 PFU	(A) PBS (n=6)<br>(B) PBS+ empty virus (n=6)<br>(C) PBS+ VEGF virus (n=6)<br>(D) PBS+ eNOS virus (n=6)	Capillary density<br><br>Protein expression<br><br>Effect of inhibition by indomethacin	7 days	dorsal random-pattern skin flap, 4x10cm	24 Sprague-Dawley male rats (350-375g)	Syringe fitted with a 30-gauge needle and injected subdermally into the distal half of the skin flap 7 days before surgery. The injections were spaced 0.5 cm apart along both sides of the midline, at 1 cm from the midline.	Capillary density (vessels/mm2) 7h prior to elevation:<br>(A) 20<br>(B) 18<br>(C) 30 (P<0.05)<br><br>Skin blood flow (ml/min) 9h post elevation:<br>(A) 0.21±0.04<br>(B) 0.20±0.02<br>(C) 0.43±0.06

										$P<0.05$
Rah; 2014	HGF	Enhancement of flap survival	1x10^{7} PFU	(A) HGF-virus (n=10)<br>(B) 500ng recombinant HGF (n=10)<br>(C) PBS (n=10)	Ratio of blood flow<br><br>CD31-positive vessels count<br><br>VEGF expression	10 days	Dorsal skin flap with panniculus carnosus, 3x9cm	30 Sprague-Dawley male rats (300-350g)	Injections made into the subdermal layer of the entire area of the skin flap (8 injections) 2 days before flap elevation and immediately after flap elevation.	Blood flow (perfusion units):<br>Mid-distal flap (7/10 day post-op):<br>(A) - 0.56±0.27 0.71±0.35<br>P=0.028<br><br>Distal flap (3/7day post-op):<br>(A) - 0.56±0.50<br>0.35±0.47<br>P = 0.017<br><br>CD31-positive vessels count (10 days post-op):<br>(A) - 8.7±2.91<br>(B) – 5.8±0.80<br>(C) – 3.8±0.76<br>P = 0.037 for (A) vs. (B/C)
de Freitas; 2010	VEGF-165	Stimulation of neovascularization in flap	100 µg	(A) TRAM flap (n=8)<br>(B) TRAM flap + abdominoplasty + PBS (n=8)<br>(C) TRAM +abdominoplasty + empty plasmid (n=8)<br>(D) TRAM +abdominoplasty + VEGF plasmid (n=8)	Neovascularization	5 days	Transverse rectus abdominis musculocutaneous flap (TRAM), 3x5cm	32 Wistar male rats (350-400g)	Into fascia. Flap was constructed 30 days after abdominoplasty. During abdominoplasty 4x25ug plasmid treatment, electroporation soon after.	Mean number of vessels (in field):<br>(A) – 3.91±1.80<br>(B) - 1.76±1.28<br>(C) - 1.81±1.06 (D) - 4.70±1.99<br>P<0.01 for (D) vs. (B/C) but not (A)
Yang; 2005	VEGF-121	Increasing skin flap survival	80 µg	(A) VEGF plasmid (n=10)<br>(B) empty plasmid (n=10)<br>(C) saline (n=10	Flap survival<br>Gene expression<br>Protein expression<br>Vascular density<br>RBC in the flap (SPECT)	7 days	McFarlane flap,8x2cm	30 Sprague-Dawley female rats (280-320g)	Intramuscular (directly into the panniculus carnosus of the flap in 2 sites per flap).	RBC count (x10^{4})<br>(A) 9.46±0.87<br>(B) 4.28± 0.56<br>(C) 3.96±0.42<br>P<0.01 (A)vs(B/C)<br><br>VEGF expression<br>(A) 1.73±0.14<br>(B) 0.75±0.08<br>(C) 0.73±0.07 P<0.01 (A)vs(B/C)<br><br>VEGF protein<br>(A) 1.24±0.18<br>(B) 0.83±0.15

										(C) 0.81±0.13<br>P<0.01(A)vs(B/C)<br><br>Vessel number<br>(A) 276±30<br>(B) 154±25<br>(C) 148±27<br>P<0.01(A)vs(B/C)<br><br>Vessel density<br>(A) 0.044±0.005<br>(B) 0.021±0.002<br>(C) 0.019±0.002 P<0.01(A)vs(B/C)
Zacchigna; 2005	VEGF-165	Increasing flap survival	1.5 x 10^{11} PFU	Experiment 1<br>Epigastric flap (n=4 each)<br>(A) VEGF intra-op<br>(B) VEGF 7d pre-op<br>(C) VEGF14d pre-op<br>(D) LacZ intra-op<br>(E) LacZ 7d pre-op<br>(F) LacZ 14d pre-op<br><br>TRAM flap (n=4 each)<br>(G) VEGF, intra-op<br>(H) VEGF, 7d pre-op<br>(I) VEGF, 14d pre-op<br>(J) LacZ, intra-op<br>(K) LacZ, 7d pre-op<br>(L) LacZ 14d pre-op<br><br>Experiment 2<br>TRAM (n=20 each)<br>(AA) VEGF, 14d pre-op<br>(BB) lacZ, 14d pre-op	HE assessment (2nd exp)	Exp 1:<br>7 days<br><br>Exp 2:<br>7 days<br>(24 animals)<br>22 days<br>(16 animals	Epigastric flap, 5x8cm<br><br>TRAM flap, 5x8cm	88 Wistar male rats (250-300g)	Direct 10 equally-spaced subcutaneous (epigastric flap) or intramuscular injection near perforator(TRAM flap).	Histology – in semi quantitative analysis (AA) flaps showed improved skin tissue quality (total score 11.9 vs 6.3 in (BB) vs 14.8 in normal skin)<br><br>CD31 – AA vs BB approx. 145 vs 60<br>(no of vessels)<br>a-SMA 45 vs 15 (no of arteries)<br>alleged p-values, p<0.05, p<0.05
Yi; 2006	VEGF-165	Increasing flap survival	5 x 10^5 cells	(A) transfected cells (n=10<br>(B) non-transfected cells (n=10)<br>(C) culture medium (n=10)	In vitro:<br>MTT Assay<br><br>In vivo:	28 days	cranially based flap, with the flap base 0.5 cm caudal to the	30 athymic nude mice, 8-16 week old	Subcutaneous injection of 0,5 ml 3 days prior to flap elevation.	MTT Assay:<br>(A) 0.42±0.02<br>(B) 0.31±0.01 P<0.05<br>Adhesion:<br>(A) 12.7±0.6

					Plasma VEGF levels<br><br>Perfusion		occipital neck line, 1.25x2.5 cm			(B) 4.5±0.4<br>P<0.05<br><br>In vivo:<br>serum VEGF lvl<br>(A) vs. (B) (345;581,432,210,30 pg/ml vs 30,25, 28, ,23, 20); significantly in 1,4,7 and 14 days (P<0.05)<br><br>Flap perfusion<br>(A)>(B)>(C) significant differences in days 4,7,14,28<br><br>Capillaries/mm2<br>(A) 37<br>(B) 30<br>(C) 15<br>P<0.05 for (A) vs.(B) and (B)vs. (C)
Spanholtz; 2009	VEGF-165	Increasing flap survival	1 x 10^7 cells	(A1) VEGF-FB, 14d prior flap elevation (n=5)<br>(A2) VEGF-FB, 7d prior flap elevation (n=5)<br>(A3) VEGF-FB, 0d prior flap elevation (n=10)<br>(B1) GFP-FB,14d pre-flap elevation (n=5)<br>(B2) GFP-FB, 7d pre-flap elevation (n=5)<br>(B3) GFP-FB, 0d prior flap elevation (n=10)<br>(C1) FB (nonmodified), 14d prior flap elevation (n=5)<br>(C2) FB (nonmodified), 7d prior flap elevation (n=5)<br>(C3) FB (nonmodified), 0d prior flap elevation (n=5)<br>(D1) only medium, 14d prior flap elevation (n=5)	In vitro:<br>VEGF expression<br><br>In vivo:<br>Flap survival<br><br>Blood vessels quantity (histology and anti-CD31 IHC)	7 days	McFarlane flap, 2x8 cm	80 Sprague-Dawley female rats (200-225g)	Different elevation times after injections – 14, 7, 0 (intra-OP) days prior flap elevation. 10 or 20 locations: 10 locations within the flap and 10 locations in the surrounding wound margin – A (flap alone), B (flap+surrounding), therefore A1A or A1B Each injection delivered 5x10^5 cells in 0.05 mL.	Blood vessel count (n/HPF):<br>(A1A) 11<br>(A1B) 10<br>(A2A) 13<br>(A2B) 17<br>(A3A) 10<br>(A3B) 11<br>(B?) 11<br>(C?) 11<br>(D?) 10<br>P<0.01 for (A2B) vs. all other

				(D2) only medium, 7d prior flap elevation<br>(D3) only medium, 0d prior flap elevation						
Spanholtz; 2011	VEGF-165 bFGF	Increasing ischemic/non-ischemic flap survival	5 x 10^6 cells	(A1A) bFBF + VEGF FB into flap alone (F), 14d prior flap elevation (PFE) (n=15)<br>(A1B) bFBF + VEGF FB into flap+surrounding (F+S), 14d PFE (n=15)<br>(A2A) bFBF + VEGF FB F, 7d PFE (n=15)<br>(A2B) bFBF + VEGF FB, F+S, 7d PFE (n=15)<br>(B1A) bFGF FB, F, 14d PFE (n=15)<br>(B1B) bFGF FB, F+S, 14d PFE (n=15)<br>(B2A) bFGF FB, F, 7d PFE n=15)<br>(B2B) bFGF FB, F+S, 7d PFE (n=15)<br>(C1A) non-modified FB, F, 14d PFE (n=15)<br>(C1B) non-modified FB, F+S, 14d PFE (n=15)<br>(C2A) non-modified FB, F, 7d PFE (n=15)<br>(C2B) non-modified FB, F+S, 7d PFE (n=15)<br>(D1A) pAdcos45.GFP FB, F, 14d PFE (n=15)<br>(D1B) pAdcos45.GFP FB, F+S, 14d PFE (n=15)<br>(D2A) pAdcos45.GFP FB, F, 7d PFE (n=15)<br>(D2B) pAdcos45.GFP FB, F+S, 7d PFE (n=15)<br>(E1A) DMEM, F, 14d PFE (n=15)<br>(E1B) DMEM, F+S, 14d PFE (n=15)<br>(E2A) DMEM, F, 7d PFE (n=15)<br>(E2B) DMEM, F+S, 7d PFE (n=15)	Histology	14 days	McFarlane flap, 2x8 cm	320 Sprague-Dawley female rats (200-225g)	40 injection sites:, 20 within flap (A – flap alone), 20 in flap surrounding (B -both), subdermal injections, 1 or 2 weeks before flap elevation.	Blood vessel density (/HPF)<br>(A2B) 43**<br>(A2A) 37**<br>(A1B) 33**<br>(A1A) 24<br>(B2B) 29*<br>(B2A) 17<br>(B1B) 24*<br>(B1A) 21*<br>(C/D/E) 5-7<br><br>**P<0.001<br>*P < 0.05 vs. controls<br><br>SMA staining<br>7-9/HPF<br>Statistically significantly higher no of arterial vessels in groups (A2B), (A2A), (A1B) - (9,7,7)<br>P<0.05

Jafari; 2017	HGF	Increasing ischemic flap survival	25 µg	(A) HGF plasmid, 24 hours before surgery (n=5)<br>(B) HGF plasmid, 24 hours after surgery (n=5)<br>(C) no treatment (n=5)	Flap necrosis (planimetry)<br><br>Doppler<br><br>IHC (qHGF)	7 days	McFarlane flap, 3x9cm	15 Wistar male rats (290-320g)	4 sites of intradermal injections (25µl each) 3 located in the midline within flap, 1 outside ; 8 pulses of 200 V/cm (for 10 msec) using a pulse generator (BTX Gemini X2 System).	Laser Index<br>(A) 57.27±24.65<br>(B) 48.98±4.70<br>(C) 33.96±10.92<br><br>P=0.0317 for (A) vs. (C)<br>P=0.0159 for (B) vs. (C)<br><br>Semiquantitative histology - inflammatory cell score,<br>(A) 1.40±0.15<br>(B) 1.73±0.60<br>(C) 1.80±0.18<br>P = 0.0317 for (A) vs. (C)<br><br>CD31+ vessel density (vessels/mm2)<br>(A) 3.46±0.81<br>(B) 3.31±0.40<br>(C) 1.73±0.62<br>P=0.0079 for (B) vs. (C)<br>P=0.0159 for (A) vs. (C)<br><br>HGF IHS optical density:<br>(A) 2.91±0.05<br>(B) 2.87±0.15<br>(C) 2.42±0.16<br>P=0.0079 for (A) vs. (C)<br>P=0.0079 for (B) vs. (C)

Table S4. Multivariate meta-regression model for secondary outcome analysis.

Variable	Effect estimate (95% confidence interval)	p-value
**Assessment time**	**1.00 (0.38 to 1.63)**	**0.0017**
Administration time	0.06 (-0.14 to 0.25)	0.57
HGF (intervention) [a]	0.48 (-4.95 to 5.92)	0.86
**VEGF (intervention) [a]**	**4.85 (0.08 to 9.62)**	**0.046**
VEGF + FGF (intervention) [a]	2.68 (-0.14 to 0.25)	0.57

[a] – reference: FGF (intervention)

Figure S17. Funnel plot regarding publication bias showing secondary outcome interventions.

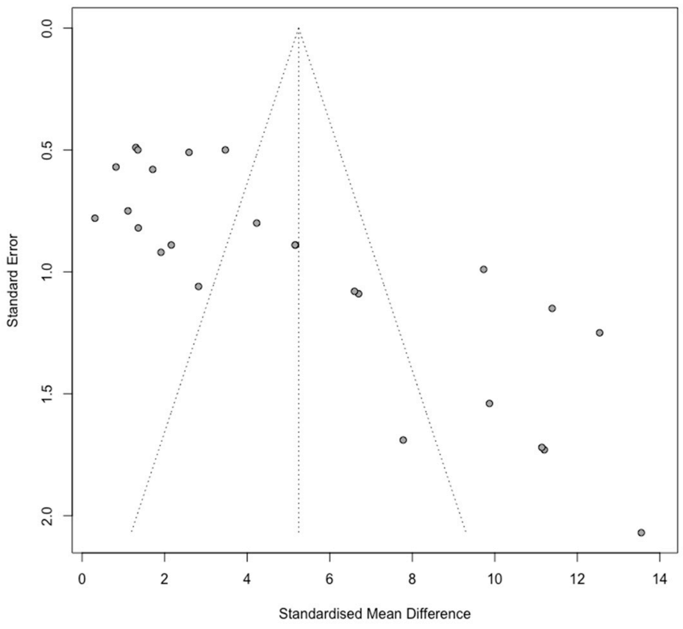

<u>Secondary outcome – vessels density</u>

intercept	95% CI	t	p
7.749	5.1 – 10.4	5.739	7.616578e-06

Eggers' test indicates the presence of funnel plot asymmetry.

Table S5. Table presenting included studies with risk of bias scores, assessed based on SYRCLE Risk of Bias Tool For Animal Studies.

First author, date	1	2	3	4	5	6	7	8	9	10	SUM (Y=1, N=0)
**MUSCLE FLAPS**											
Neumeister, 2001	N	Y	N	N	N	N	N	Y	Y	Y	4
de Freitas, 2010	Y	Y	N	N	N	N	N	N	Y	Y	4
Hijjawi, 2004	N	Y	N	N	N	N	N	Y	Y	N	3
Zhang, 2005	N	Y	N	N	N	Y	N	Y	Y	Y	5
Rezende, 2010	Y	Y	N	N	N	N	N	Y	Y	N	4
Antonini, 2007	N	Y	N	N	N	N	N	N	N	N	1
Zacchinga, 2005	N	Y	N	N	N	N	N	Y	N	N	2
**MCFARLANE FLAPS**											
Liu, 2005 (Liu1)	N	Y	N	N	N	Y	N	Y	Y	Y	5
Liu, 2004	N	Y	N	N	N	Y	N	Y	Y	Y	5
Liu, 2005 (Liu2)	Y	Y	N	N	N	Y	N	Y	Y	Y	6
Holzbach, 2010	Y	Y	N	N	N	N	N	N	Y	Y	4
Nakagawa, 2007	N	Y	N	N	N	N	N	Y	Y	N	3
Fujihara, 2005	N	Y	N	N	N	Y	N	Y	Y	Y	5
Ferraro, 2009	N	Y	N	N	N	N	N	Y	Y	Y	4
Chang, 2021	N	Y	N	N	N	Y	N	Y	Y	Y	5
Basu, 2014	N	Y	N	N	N	N	N	Y	Y	N	3
Yang, 2005	Y	Y	N	N	N	Y	N	Y	Y	Y	6
Wang, 2006	N	Y	N	N	N	N	N	Y	Y	Y	4
Jafari, 2017	Y	Y	N	N	N	N	N	Y	Y	Y	5
Jafari, 2018	N	Y	N	N	N	Y	N	Y	Y	Y	5
Jafari, 2021	Y	Y	N	N	N	Y	N	Y	Y	Y	6
Huang, 2006	N	Y	N	N	N	N	N	Y	Y	Y	4
Rah, 2014	N	Y	N	Y	N	N	N	Y	Y	Y	5

Liu, 2009	Y	Y	N	N	N	N	N	Y	Y	Y	5
Lee, 2011	N	Y	N	N	N	N	N	Y	Y	Y	4
Choi, 2020	N	Y	N	N	N	N	N	Y	Y	Y	4
Lou, 2021	Y	Y	N	N	N	Y	N	Y	Y	Y	6
Gurunluoglu, 2005	N	Y	Y	N	N	N	N	Y	Y	Y	5
Giunta, 2005	N	Y	Y	N	N	N	N	Y	Y	Y	5
Wang, 2011	N	Y	N	N	N	N	N	Y	Y	Y	4
Wang, 2013	N	Y	N	N	N	N	N	Y	Y	Y	4
Chen, 2011	Y	Y	Y	N	N	Y	Y	Y	Y	Y	8
Rinsch, 2001	N	Y	N	N	N	N	N	N	Y	Y	3
Yi, 2006	N	Y	N	N	N	N	Y	Y	Y	Y	5
Zheng, 2008	Y	Y	Y	N	N	N	Y	Y	Y	Y	7
Spanholtz, 2009	N	Y	N	N	N	N	N	N	Y	N	2
Spanholtz, 2011	N	Y	N	N	N	N	N	N	Y	N	2
Luo; 2021	Y	Y	N	N	N	N	N	N	N	N	1
**EPIGASTRIC FLAPS**											
Michlits, 2007	N	Y	Y	N	N	N	N	Y	Y	Y	5
Taub, 1998	N	Y	N	N	N	N	N	Y	Y	Y	4
O'Toole, 2002	N	Y	N	N	N	N	N	Y	Y	Y	4
Meirer, 2007	N	Y	N	N	N	N	Y	Y	Y	Y	5
Lubiatowski, 2002	N	Y	N	N	N	N	N	Y	Y	Y	4
Huemer, 2004	N	Y	Y	N	N	N	N	Y	Y	Y	5
Huemer, 2005	N	Y	N	N	N	N	N	Y	Y	N	3
Jung, 2003	N	Y	Y	N	N	N	N	Y	Y	Y	5
Gurunluoglu, 2002	N	Y	N	N	Y	N	N	Y	Y	Y	5
Machens, 2002	N	Y	Y	N	N	N	N	Y	Y	N	4
Uemura, 2012	N	Y	N	N	N	N	N	Y	Y	Y	4
Fu-Gui; 2011	Y	Y	N	N	N	N	Y	Y	Y	Y	6

	**37**	0	**42**	**49**	**49**	**39**	**45**	7	3	11	**4,34**

Table S6. Table presenting summary of findings (SoF) with regards to GRADE Approach for VEGF intervention group.

**VEGF treatment vs control**[1]			
**Patients or population:**[2] Laboratory rats (varied strains)<br>**Settings:**[b] Varied laboratories around the world<br>**Intervention:**[b] VEGF gene therapy delivery<br>**Comparison:**[b] Sham gene therapy delivery			
**Outcomes**[3]	**Overall improvement (standarised/ non-standarsied mean difference)**[4]	**Number of participants (Studies)**[5]	**Certainty of the evidence (GRADE)***[6]
**Flap survival**	15.66% (95% CI 11.80 – 19.52) increase with intervention	927 rats<br>(27 studies)	⊕⊕⊖⊖<br>Low
**Vessel density**	4.80 SMD (95% CI 2.41 – 7.18) increase with intervention	unspecified<br>(10 studies)	⊕⊕⊖⊖<br>Low
* GRADE Working Group grades of evidence<br>**High** = This research provides a very good indication of the likely effect. The likelihood that the effect will be substantially different[†] is low.<br>**Moderate** = This research provides a good indication of the likely effect. The likelihood that the effect will be substantially different[†] is moderate.<br>**Low** = This research provides some indication of the likely effect. However, the likelihood that it will be substantially different[†] is high.<br>**Very low** = This research does not provide a reliable indication of the likely effect. The likelihood that the effect will be substantially different[†] is very high.<br>[†] Substantially different = a large enough difference that it might affect a decision			

[1] A title indicating the comparison summarised in the table

[2] The characteristics of the evidence, including the types of participants (patients or populations), types of settings (e.g. countries) where the studies were done, the intervention and what the intervention was compared to

[3] The most important outcomes, including the intended benefits, possible harms and costs

[4] The estimated impact of the intervention on each outcome (preferably provided quantitatively)

[5] The amount of information upon which the information is based, such as the number of participants or units (e.g. facilities), as well as the number of studies

[6] The quality of the evidence for each outcome

VEGF treatment may improve flap survival and vessel density.

Table S7. Table presenting summary of findings (SoF) with regards to GRADE Approach for FGF intervention group.

**FGF treatment vs control**[7]			
**Patients or population:**[8] Laboratory rats (varied strains)<br>**Settings:**[b] Varied laboratories around the world<br>**Intervention:**[b] FGF gene therapy delivery<br>**Comparison:**[b] Sham gene therapy delivery			
**Outcomes**[9]	**Overall improvement (standarised/ non-standarsied mean difference)**[10]	**Number of participants (Studies)**[11]	**Certainty of the evidence (GRADE)*** [12]
**Flap survival**	3.84% (95% CI 1.13 – 6.55) increase with intervention	281 rats<br>(6 studies)	⊕⊖⊖⊖<br>Very low
**Vessel density**	5.53 SMD (95% CI 2.23 – 8.84) increase with intervention	unspecified<br>(5 studies)	⊕⊖⊖⊖<br>Very low
* GRADE Working Group grades of evidence<br>**High** = This research provides a very good indication of the likely effect. The likelihood that the effect will be substantially different† is low.<br>**Moderate** = This research provides a good indication of the likely effect. The likelihood that the effect will be substantially different† is moderate.<br>**Low** = This research provides some indication of the likely effect. However, the likelihood that it will be substantially different† is high.<br>**Very low** = This research does not provide a reliable indication of the likely effect. The likelihood that the effect will be substantially different† is very high.			

[7] A title indicating the comparison summarised in the table

[8] The characteristics of the evidence, including the types of participants (patients or populations), types of settings (e.g. countries) where the studies were done, the intervention and what the intervention was compared to

[9] The most important outcomes, including the intended benefits, possible harms and costs

[10] The estimated impact of the intervention on each outcome (preferably provided quantitatively)

[11] The amount of information upon which the information is based, such as the number of participants or units (e.g. facilities), as well as the number of studies

[12] The quality of the evidence for each outcome

† Substantially different = a large enough difference that it might affect a decision

It is uncertain whether FGF treatment improves flap survival or vessel density because the certainty of the evidence is very low.

Table S8. Table presenting summary of findings (SoF) with regards to GRADE Approach for *PDGF* intervention group.

**PDGF treatment vs control**[13]			
**Patients or population:**[14] Laboratory rats (varied strains)<br>**Settings:**[b] Varied laboratories around the world<br>**Intervention:**[b] PDGF gene therapy delivery<br>**Comparison:**[b] Sham gene therapy delivery			
**Outcomes**[15]	**Overall improvement (standarised/ non-standarsied mean difference)**[16]	**Number of participants (Studies)**[17]	**Certainty of the evidence (GRADE)***[18]
**Flap survival**	13.44% (95% CI 3.53 – 23.35) increase with intervention	93 rats<br>(4 studies)	⊕⊖⊖⊖<br>Very low
* GRADE Working Group grades of evidence<br>**High** = This research provides a very good indication of the likely effect. The likelihood that the effect will be substantially different† is low.<br>**Moderate** = This research provides a good indication of the likely effect. The likelihood that the effect will be substantially different† is moderate.<br>**Low** = This research provides some indication of the likely effect. However, the likelihood that it will be substantially different† is high.<br>**Very low** = This research does not provide a reliable indication of the likely effect. The likelihood that the effect will be substantially different† is very high.<br>† Substantially different = a large enough difference that it might affect a decision<br>**It is uncertain whether PDGF treatment improves flap survival because the certainty of the evidence is very low.**			

[13] A title indicating the comparison summarised in the table

[14] The characteristics of the evidence, including the types of participants (patients or populations), types of settings (e.g. countries) where the studies were done, the intervention and what the intervention was compared to

[15] The most important outcomes, including the intended benefits, possible harms and costs

[16] The estimated impact of the intervention on each outcome (preferably provided quantitatively)

[17] The amount of information upon which the information is based, such as the number of participants or units (e.g. facilities), as well as the number of studies

[18] The quality of the evidence for each outcome

Table S9. Table presenting summary of findings (SoF) with regards to GRADE Approach for HGF/HGF+PGIS intervention group.

**HGF or HGF+PGIS treatment vs control**[19]			
**Patients or population:**[20] Laboratory rats (varied strains)<br>**Settings:**[b] Varied laboratories around the world<br>**Intervention:**[b] HGF or HGF+PGIS gene therapy delivery<br>**Comparison:**[b] Sham gene therapy delivery			
**Outcomes**[21]	**Overall improvement (standarised/ non-standarsied mean difference)**[22]	**Number of participants (Studies)**[23]	**Certainty of the evidence (GRADE)***[24]
**Flap survival**	5.61% (95% CI 0.43 – 10.78) increase with intervention	135 rats<br>(3 studies)	⊕⊖⊖⊖<br>Very low
**Vessel density**	1.64 SMD (95% CI 0.84 – 2.43) increase with intervention	unspecified<br>(2 studies)	⊕⊖⊖⊖<br>Very low
* GRADE Working Group grades of evidence<br>**High** = This research provides a very good indication of the likely effect. The likelihood that the effect will be substantially different† is low.<br>**Moderate** = This research provides a good indication of the likely effect. The likelihood that the effect will be substantially different† is moderate.<br>**Low** = This research provides some indication of the likely effect. However, the likelihood that it will be substantially different† is high.<br>**Very low** = This research does not provide a reliable indication of the likely effect. The likelihood that the effect will be substantially different† is very high.<br>† Substantially different = a large enough difference that it might affect a decision<br>**It is uncertain whether HGF or HGF+PGIS treatment improves flap survival or vessel density because the certainty of the evidence is very low.**			

[19] A title indicating the comparison summarised in the table

[20] The characteristics of the evidence, including the types of participants (patients or populations), types of settings (e.g. countries) where the studies were done, the intervention and what the intervention was compared to

[21] The most important outcomes, including the intended benefits, possible harms and costs

[22] The estimated impact of the intervention on each outcome (preferably provided quantitatively)

[23] The amount of information upon which the information is based, such as the number of participants or units (e.g. facilities), as well as the number of studies

[24] The quality of the evidence for each outcome

Table S10. Table presenting summary of findings (SoF) with regards to GRADE Approach for VEGF+FGF intervention group.

**VEGF + FGF treatment vs control**[25]			
**Patients or population:**[26] 140 rats (2 studies)<br>**Settings:**[b] Varied laboratories around the world<br>**Intervention:**[b] VEGF + FGF gene therapy delivery<br>**Comparison:**[b] Sham gene therapy delivery			
**Outcomes**[27]	**Overall improvement (standarised/ non-standarsied mean difference)**[28]	**Number of participants (Studies)**[29]	**Certainty of the evidence (GRADE)*** [30]
**Flap survival**	8.64% (95% CI 6.94 – 10.34) increase with intervention	140 rats<br>(2 studies)	⊕⊖⊖⊖<br>Very low
**Vessel density**	8.28 SMD (95% CI 4.08 – 12.48) increase with intervention	unspecified<br>(2 studies)	⊕⊖⊖⊖<br>Very low
* GRADE Working Group grades of evidence<br>**High** = This research provides a very good indication of the likely effect. The likelihood that the effect will be substantially different† is low.<br>**Moderate** = This research provides a good indication of the likely effect. The likelihood that the effect will be substantially different† is moderate.<br>**Low** = This research provides some indication of the likely effect. However, the likelihood that it will be substantially different† is high.<br>**Very low** = This research does not provide a reliable indication of the likely effect. The likelihood that the effect will be substantially different† is very high.<br>† Substantially different = a large enough difference that it might affect a decision<br>**It is uncertain whether VEGF + FGF treatment improves flap survival or vessel density because the certainty of the evidence is very low.**			

25 A title indicating the comparison summarised in the table
26 The characteristics of the evidence, including the types of participants (patients or populations), types of settings (e.g. countries) where the studies were done, the intervention and what the intervention was compared to
27 The most important outcomes, including the intended benefits, possible harms and costs
28 The estimated impact of the intervention on each outcome (preferably provided quantitatively)
29 The amount of information upon which the information is based, such as the number of participants or units (e.g. facilities), as well as the number of studies
30 The quality of the evidence for each outcome
